# SYT12 plays a critical role in oral cancer and may be a novel therapeutic target

**DOI:** 10.7150/jca.32582

**Published:** 2019-08-27

**Authors:** Keitaro Eizuka, Dai Nakashima, Noritoshi Oka, Sho Wagai, Toshikazu Takahara, Tomoaki Saito, Kazuyuki Koike, Atsushi Kasamatsu, Masashi Shiiba, Hideki Tanzawa, Katsuhiro Uzawa

**Affiliations:** 1 Department of Oral Science, Graduate School of Medicine, Chiba University, Chiba, Japan; 2 Department of Dentistry and Oral-Maxillofacial Surgery, Chiba University Hospital, Chiba, Japan; 3 Department of Medical Oncology, Graduate School of Medicine, Chiba University, Chiba, Japan

**Keywords:** Oral squamous cell carcinoma, SYT12, CAMK2N1, CAMK2, L-dopa

## Abstract

Synaptotagmin12 (SYT12) has been well characterized as the regulator of transmitter release in the nervous system, however the relevance and molecular mechanisms of SYT12 in oral squamous cell carcinoma (OSCC) are not understood. In the current study, we investigated the expression of SYT12 and its molecular biological functions in OSCC by quantitative reverse transcriptase polymerase chain reaction, immunoblot analysis, and immunohistochemistry. SYT12 were up-regulated significantly in OSCC-derived cell lines and primary OSCC tissue compared with the normal counterparts (P<0.05) and the SYT12 expression levels were correlated significantly with clinical indicators, such as the primary tumoral size, lymph node metastasis, and TNM stage (P<0.05). SYT12 knockdown OSCC cells showed depressed cellular proliferation, migration, and invasion with cell cycle arrest at G1 phase. Surprisingly, we found increased calcium/calmodulin-dependent protein kinase 2 (CAMK2) inhibitor 1 (CAMK2N1) and decreased CAMK2-phosphorylation in the knockdown cells. Furthermore, treatment with L-3, 4-dihydroxyphenylalanine (L-dopa), a drug approved for Parkinson's disease, led to down-regulation of SYT12 and similar phenotypes to SYT12 knockdown cells. Taken together, we concluded that SYT12 plays a significant role in OSCC progression via CAMK2N1 and CAMK2, and that L-dopa would be a new drug for OSCC treatment through the SYT12 expression.

## Introduction

Oral cancer is the sixth most prevalent form of cancer 1. Oral squamous cell carcinomas (OSCCs), comprising about 90% of all oral cancer 2, are the highest morbidity of all head and neck cancers 3. More than 500,000 new cases and 300,000 deaths annually were reported 4. Although great progress has been made for the treatment and diagnosis, the 5-year survival rates for patients with OSCCs remain unchanged at 50% to 55% from nearly 5 decades ago 5. Therefore, understanding the molecular mechanisms underlying OSCC development and progression is necessary for improving the prognoses.

Synaptotagmins (SYTs) are membrane proteins comprised of a short N-terminal noncytoplasmic sequence, a single transmembrane region, a linker sequence of varying lengths, and two C2 domains. The SYTs were discovered because they are the Ca^2+^ sensor for neurotransmitter release in presynaptic nerve terminals, and SYT1 was identified first 6. Eight of these SYTs have C2 domains that bind to Ca^2+^ (SYT1-3, 5-7, 9, and 10) while the other eight do not (Syt4, 8, and 11-16) 7. The *SYT12* gene encodes a family of proteins involved in regulating transmitter release in the nervous system 8,9.

A recent study reported that SYT12 overexpression was correlated with metastasis and SYT12 was a biomarker that tended to predict greater disease progression in patients with papillary thyroid cancer 10. However, the molecular biologic role of SYT12 in cancer remains unknown. In the current study, we explain for the first time the novel molecular mechanism of SYT12 in OSCCs and evaluate a new candidate for medical treatment of OSCCs.

## Materials and Methods

### Ethics statement

The ethical committee of the Graduate School of Medicine, Chiba University, approved the research protocol (protocol number 680). All patients provided written informed consent before inclusion in the study.

### Cells and tissue samples

Eleven human OSCC-derived cell lines (HSC-2, HSC-3, HSC-3-M3, HSC-4, Sa3, Ca9-22, KOSC-2, SAS, SAS-H1, Ho-1-u-1, and Ho-1-N-1) were purchased from RIKEN BioResource Center (Tsukuba, Ibaraki, Japan) and the Japanese Collection of Research Bioresources Cell Bank (Ibaraki, Osaka, Japan) 11-13. We obtained human normal oral keratinocyte (HNOKs) from young healthy patients and cultured the cells as described previously 14-17. The cells used in this study were within 10 passages from thawing. They also were routinely tested using an EZ-PCR Mycoplasma Test Kit (Biological Industries, Kibbutz Beit Haemek, Israel).

### mRNA expression analysis

Total RNA was prepared using TRIZOL reagent (Invitrogen, Carlsbad, CA, USA). Quantitative reverse transcriptase-polymerase chain reaction (qRT-PCR) was carried out as described previously 11,18 with the following primers. The sequences of the gene-specific primers were as follows: *SYT12* (5′-CGAAGCCATGATCTTCTCG*-*3' and 5'-GCTCTCAGCCACCGTCAC-3') and *glyceraldehyde-3-phosphate dehydrogenase* (*GAPDH*) (5′-AGCCACATCGCTCAGACAC-3′ and 5′-GCCCAATACGACCAAATCC-3′).

### Reagents

L-dopa was purchased from Cayman Chemical (Ann Arbor, MI, USA). The following antibodies were used: anti-SYT12 (M519) (Merck & Co., Inc., Kenilworth, NJ, USA); anti-CAMK2N1 (PA5-23740) (Invitrogen, CA, USA); anti-CAMK2 (ab52476) and anti-CAMK2 (Phospho T286) (ab32678) (abcam, Tokyo, Japan); anti-p27^Kip1^ (#3686) and anti-CDK2 (#2546) (Cell Signaling Technology, Danvers, MA, USA); anti-cyclin E (sc-377100), and GAPDH (sc-3223) (Santa Cruz Biotechnology, Santa Cruz, CA, USA).

### Immunoblotting

Immunoblotting was performed as previously reported 15,19 with each appropriate antibody mentioned previously.

### Immunohistochemistry (IHC)

IHC and calculation of the IHC score were performed as described previously 15,19-21, with appropriate antibody, anti-SYT12 (1:100 dilution), at 4°C. SYT12 was classified as positive and negative using the median score of all tumors as the cut-off points 22.

### Transfection with shRNA plasmid

We established stable knockdown transfectants, and the cell lines (HSC-3, SAS) were transfected with shRNA targeting (shSYT12) (sc-96796) and control shRNA (shMock) (sc-108060) (Santa Cruz Biotechnology, Santa Cruz, CA, USA) using Lipofectamine 3000 (Invitrogen, CA, USA) according to the manufacturer's protocol. The stable transfectants were isolated in a culture medium containing puromycin (2 µg/ml) (Santa Cruz Biotechnology, Santa Cruz, CA, USA). Four weeks after transfection, a small colony was viable. The cell colonies were picked, transferred to six-well plates, and expanded gradually to 10-cm dishes. To assess the efficiency of SYT12 knockdown, we performed RT-qPCR and immunoblotting.

### Functional assays

A proliferation assay, invasion assay, and migration assay were performed as described previously 22-30.

### Cellular viability assay

Cellular viability was measured using the MTS assay (Promega, Madison, WI, USA) 30. MTS assays with cells (HSC-3, SAS) (5 × 10^3^) were performed in 96-well plates. After treatment with L-dopa (concentrations, 0, 50, 100, 200, 400, and 800 µM) for 24 h, we measured the 490-nm absorbance using a Benchmark Plus Microplate Reader (Bio-Rad, Hercules, CA, USA).

### Cell cycle analysis

Cell cycle analyses were performed as described previously 25,27,31. shRNA transfected cells and L-dopa-treated cells were examined.

### L-dopa treatment

To determine the appropriate concentration of L-dopa for OSCC cells, we performed the MTS assay, qRT-PCR, and immunoblotting. We then evaluate whether L-dopa affected the cellular proliferation and cell cycle. The cells were treated with L-dopa (50 µM) or dimethylsulfoxide as controls.

### PCR array

To identify genes that potentially interact with SYT12, we used qRT-PCR to analyze the Epithelial to Mesenchymal Transition RT^2^ Profiler PCR Array (Qiagen, Hilden, Germany).

### Statistical analysis

Each experiment was repeated at least three times, and all data are presented as the mean ± standard error of the mean. Statistical differences were analyzed using Fisher's exact test and a two-tailed, unpaired Student's *t*-test with Welch's correction for unequal variances using the BellCurve for Excel (Social Survey Research Information Co., Ltd.). P < 0.05 was considered statistically significant.

## Results

### Up-regulation of SYT12 in OSCC cell lines

**Fig 1:**
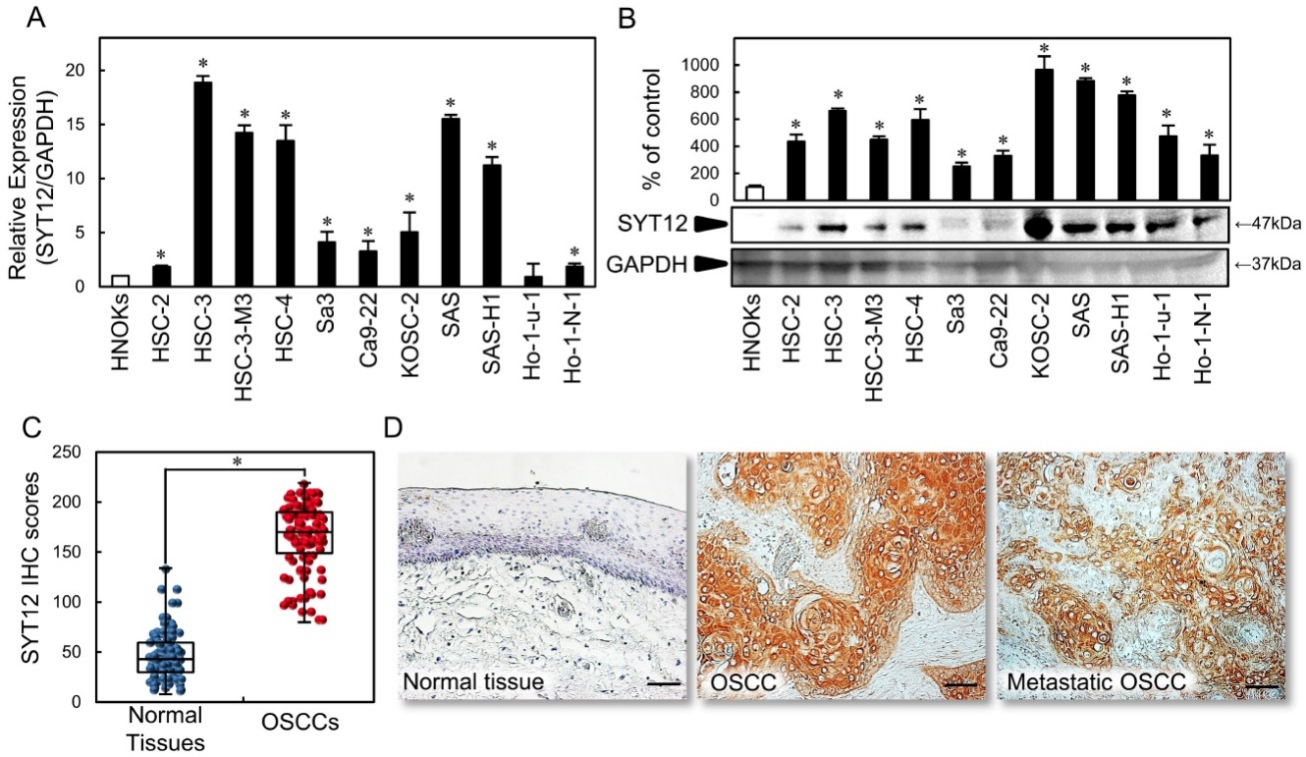
SYT12 expression in OSCC-derived cell lines and primary OSCCs. (A) Quantification of *SYT12* mRNA expression in OSCC-derived cell lines by qRT-PCR analysis (N=3). (B) Representative immunoblot analysis of SYT12 protein expression. The densitometric SYT12 protein data are normalized to GAPDH protein levels. The values are expressed as a percentage of the HNOKs (N=3). (C) The SYT12 IHC scores of the normal oral tissues and OSCCs (N=112). (D) Representative IHC results for SYT12 protein in normal tissue, primary OSCCs, and metastatic regional lymph nodes. Original magnification, x200. Scale bars, 50 μm.

To investigate the status of the *SYT12* expression as a cancer-related gene, we first conducted qRT-PCR and immunoblot analysis with 11 OSCC-derived cell lines and HNOKs. *SYT12* mRNA expression was up-regulated significantly (P < 0.05) in OSCC-derived cell lines other than Ho-1-U-1 compared with the HNOKs (Fig. 1A). Fig. 1B shows representative results of immunoblot analysis. The SYT12 protein level also increased in all OSCC cell lines compared with the HNOKs (Fig. 1B).

### Evaluation of SYT12 status in primary OSCCs

We evaluated the SYT12 expression in primary OSCCs by IHC and the IHC scoring system 22,27,31. In oral normal tissues, the SYT12 IHC scores ranged from 8.1 to 134.2 (median, 43.0), while in OSCCs the scores ranged from 80.3 to 219.0 (median, 170.2). These IHC scores in primary OSCCs were significantly (P < 0.05) higher than in normal oral tissues (Fig. 1C). Intense cytoplasmic and cell membrane staining was seen for SYT12, whereas the normal oral tissues showed negative staining. Representative IHC figures for SYT12 staining are shown (×200 magnification) (Fig. 1D). In the clinical classifications, SYT12-positive OSCCs (IHC scores > median, 170.2) were associated significantly (P < 0.05) with tumor size, regional lymph node metastasis, and the TNM stage of the OSCCs (Table 1).

### Establishment of stable SYT12 knockdown OSCC cells

**Fig 2:**
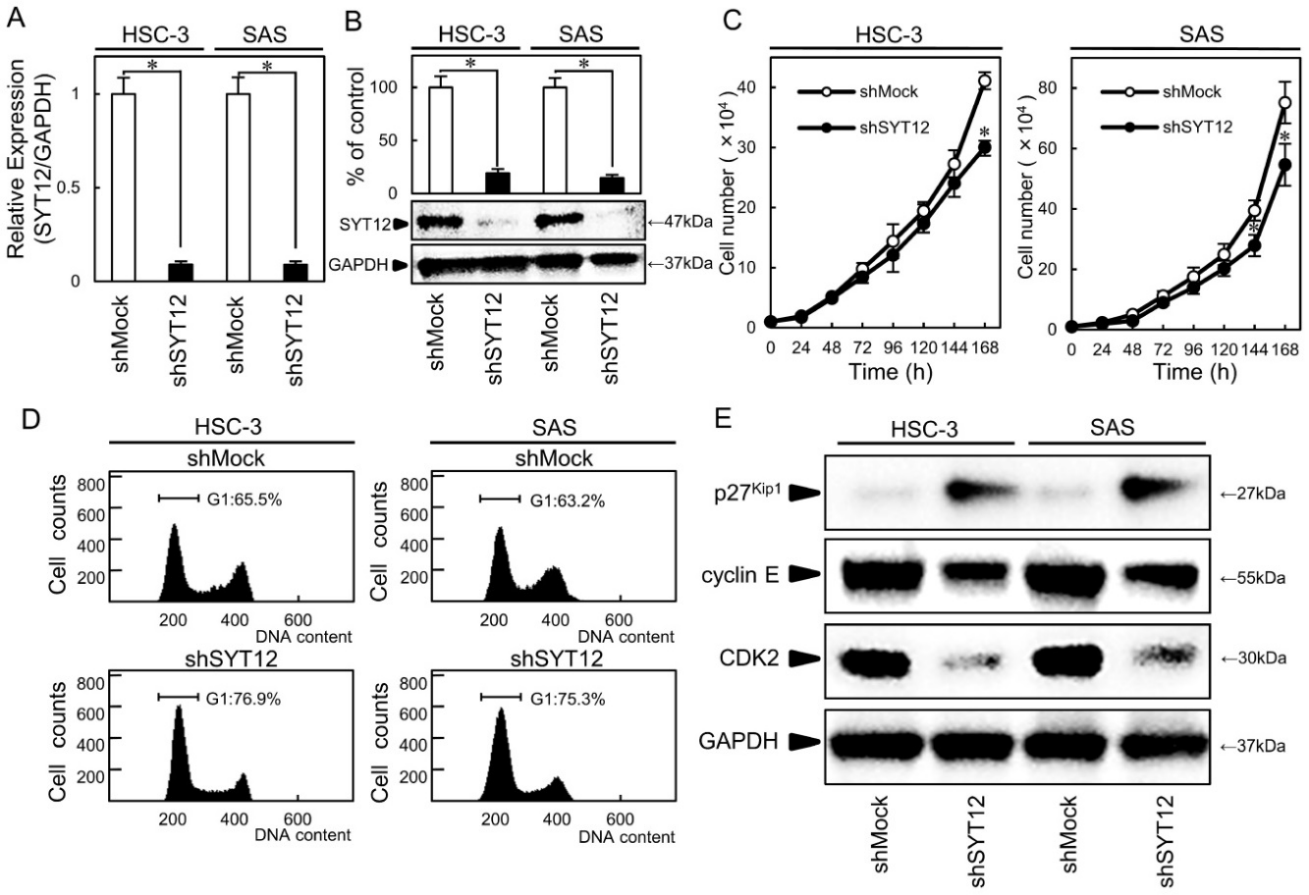
Establishment of SYT12 knockdown cells and decreased cell growth via cell cycle arrest at G1 phase. (A, B) Expression of SYT12 mRNA and protein in shMock and shSYT12 cells (HSC-3 and SAS-derived transfectants) (N=3). (C) The cellular growth of the shSYT12 cells are decreased significantly (P < 0.05) compared with the shMock cells after 7 days (168 h) (N=3). (D) A flow cytometric analysis shows that the percentage of the shSYT12 cells in the G1 phase is increased compared with the shMock cells (N=3). (E) Immunoblotting analysis shows up-regulation of p27^Kip1^ and down-regulation of cyclin E and CDK2 in the shSYT12 cells compared with the shMock cells (N=3).

HSC-3 and SAS were subjected to knockdown experiments because they had higher expression levels among the OSCC cell lines. We transfected shSYT12 and shMock vectors into the cells. To investigate the efficiency of the transfection, we performed qRT-PCR and immunoblot analysis. The SYT12 mRNA and protein expression levels in the shSYT12 cells decreased significantly (P < 0.05) compared with the shMock cells (Fig. 2A, B).

### Effect of shSYT12 cells on cellular growth and cell cycle

To evaluate the effect of SYT12 knockdown on cellular growth, a cellular proliferation assay and cell cycle analyses were performed. We found a significant (P < 0.05) decrease in cellular proliferation in the shSYT12 cells compared with the shMock cells (Fig. 2C). In the cell cycle analysis by flow cytometry, the percentage of the shSYT12 cells at the G1 phase was significantly (P < 0.05) higher than that of the shMock cells (Fig. 2D). Analysis of the G1 phase-related protein by immunoblotting showed increased p27^Kip1^ and subsequent decreased expression of cyclin E and CDK2 and in the shSYT12 cells compared with the shMock cells (Fig. 2E).

### Cellular migration and invasion assays

**Fig 3:**
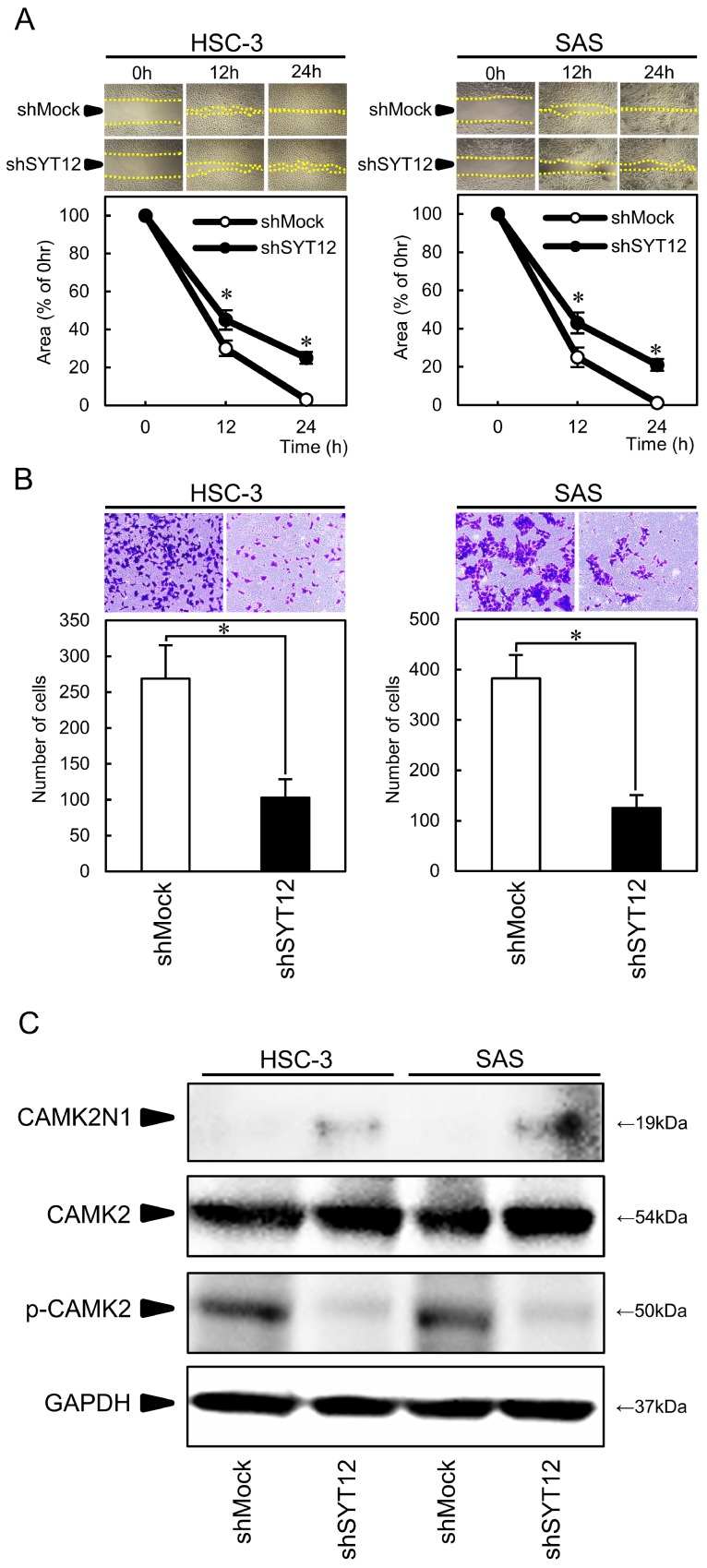
Involvement of the SYT12 signaling pathways that mediate the migratory and invasiveness phenotype of OSCCs. (A) Migration assay of shMock cells and shSYT12 cells (N=3). (B) Invasion assay of shMock cells and shSYT12 cells at 24h (N=3). (C) Immunoblot analysis shows CAMK2N1 expression is increased in the shSYT12 cells, CAMK2 expression is similar in the shMock cells and shSYT12, but phosphorylation of CAMK2 (p-CAMK2) is decreased in the shSYT12 cells compared with the shMock cells (N=3).

The migration assay showed that the area of micropipette wounds decreased significantly (P < 0.05) in shSYT12 cells after 12 h compared with the shMock cells (Fig. 3A). Analysis of the invasion assay showed significantly lower (P < 0.05) invasiveness of the shSYT12 cells compared with the shMock cells at 24 h (Fig. 3B).

### Involvement of SYT12 signaling pathways that mediated the malignant phenotype of OSCCs

To obtain the data to support the involvement of SYT12 in cancer progression, we performed PCR arrays in the shMock and shSYT12 cells. CAMK2N1 had a significantly (P<0.001) higher score (fold change, 12.4). CAMK2N1 is the endogenous inhibitor of CAMK2, which is activated by an increased intracellular Ca2+ concentration. CAMK2N1 expression was increased in the shSYT12 cells compared with the shMock cells. Furthermore, CAMK2 expression was similar in the shMock cells and shSYT12, but phosphorylation of CAMK2 decreased in the shSYT12 cells compared with the shMock cells (Fig. 3C).

### L-dopa treatment

**Fig 4:**
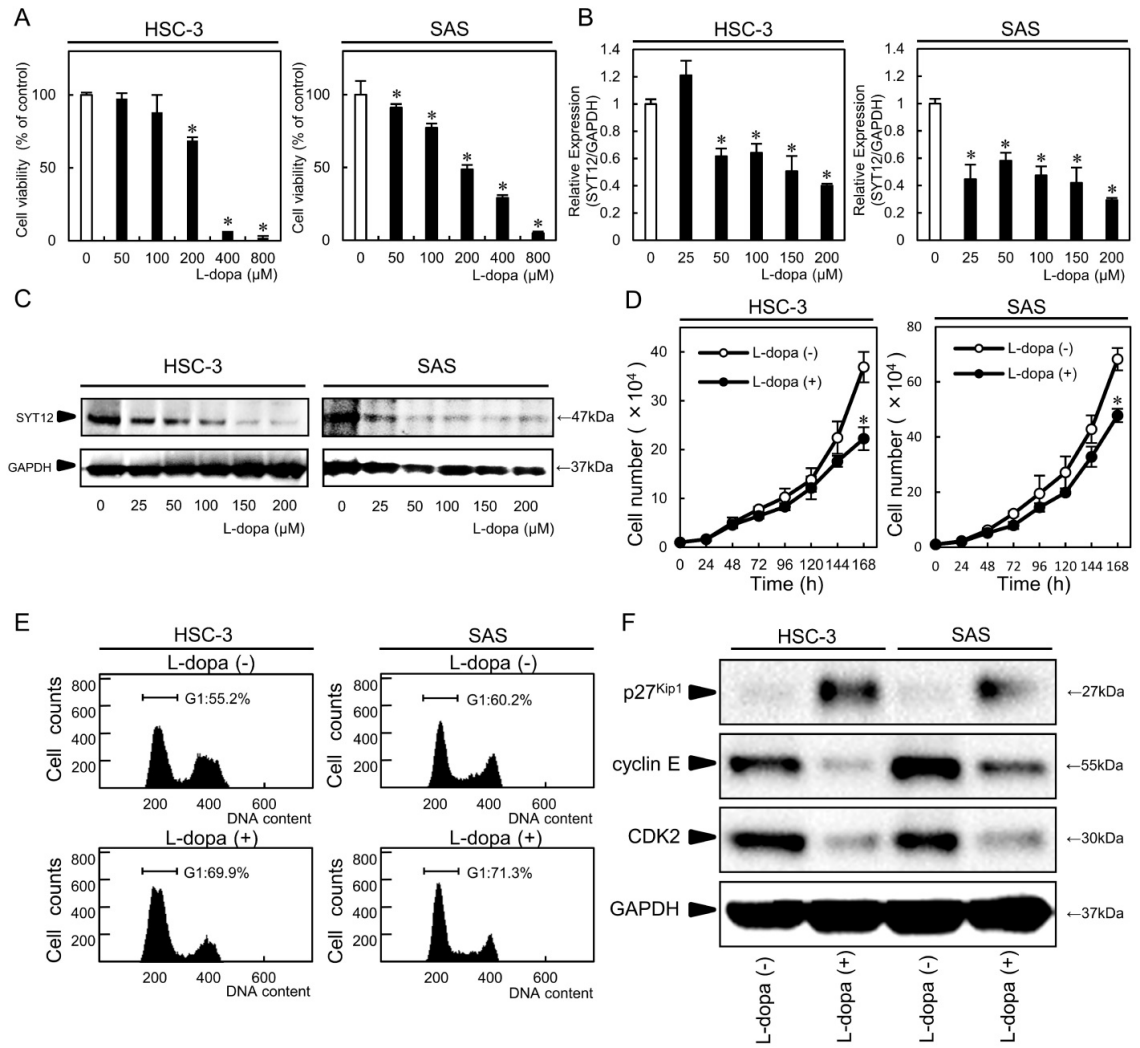
L-dopa treatment. (A) An MTS assay shows a significant decrease in cellular viability above L-dopa concentrations of 200 µM in a concentration-dependent manner (N=3). (B, C) L-dopa concentrations above 50 µM inhibit SYT12 mRNA and protein expression levels in the L-dopa-treated cells compared with controls (N=3). (D) The cellular growth of the L-dopa-treated cells is significantly (P < 0.05) lower than that of the control cells after 7 days (168 h) (N=3). (E) A flow cytometric analysis shows the percentage of L-dopa-treated cells in the G1 phase is increased compared with the control cells (N=3). (F) Immunoblot analysis shows up-regulation of p27^Kip1^ and down-regulation of CDK2 and cyclin E in the L-dopa-treated cells compared with the control cells (N=3).

We detected the SYT12 inhibitor, L-dopa, using Ingenuity Pathway Analysis. To investigate the efficiency of L-dopa for OSCC cells (HSC-3, SAS), we performed an MTS assay, which showed significantly (P<0.05) decreased cellular viability above L-dopa concentrations of 200 µM in a concentration-dependent manner (Fig. 4A). The inhibitory concentration 50s (IC_50_) for L-dopa in the HSC-3 and SAS cell lines were 259 nM and 196 nM, respectively. To verify the inhibitory effect of L-dopa on SYT12, the SYT12 expression was examined after treatment which various concentrations of L-dopa, by qRT-PCR and immunoblot analysis. L-dopa significantly (P<0.05) inhibited the SYT12 mRNA and protein expression levels above concentrations of 50 µM in both cells compared with the control cells (Fig. 4B, C). Cellular growth of the L-dopa-treated cells were significantly (P < 0.05) lower than the control cells at 144 h (Fig. 4D). Cell cycle analysis showed that the percentage of the L-dopa-treated cells at the G1 phase was significantly (P < 0.05) higher than the control cells (Fig. 4E). The addition, L-dopa-treated cells showed that the p27^Kip1^ protein expression levels were up-regulated and the cyclin E and CDK2 levels were down-regulated compared with the control cells (Fig. 4F). These results indicated that L-dopa may regulate critical functions associated with tumoral growth through down-regulation of SYT12.

## Discussion

The current results showed that SYT12 knockdown was characterized by decreased cellular growth, invasiveness, and migratory activities with CAMK2N1 overexpression and decreased CAMK2-phospholyration. In addition, L-dopa, a drug for Parkinson's disease, controlled SYT12 expression, leading to similar phenotypes of SYT12 knockdown cells.

Previous studies have shown that the SYT family genes regulate proliferation, invasion, and metastasis in several cancer cells in addition to their roles in neuronal cells 32-35. Of SYT family, SYT12 is detected as a biomarker of papillary thyroid cancer 10, but the molecular biologic function of SYT12 in other cancer cells and the interactions between SYT12 and cancer-associated signaling pathways remain unknown. To identify the SYT12-related genes, we conducted PCR array analysis using SYT12 knockdown cells and found that SYT12 affects the expression of CAMK2N1, inhibitor type1 of CAMK2, which is a known potent modulator of tumorigenesis 36. CAMK2N1 expression has tumor-suppressing roles, such as depressed cell proliferation and cell cycle arrest in certain cancers 37-39. Actually, SYT12 knockdown cells showed significant overexpression of CAMK2N1 (Fig. 3C), suggesting that down-regulation of SYT12 suppressed tumoral growth with cell cycle arrest by CAMK2N1 overexpression. CAMK2 is involved in controlling a range of cellular processes, including synaptic plasticity and memory consolidation 40,41, vascular smooth muscle polarization 42, and cellular proliferation 43. Moreover, several studies also suggest that CAMK2 regulates metastasis in several cancers 44-46. The biologic properties of CAMK2 are controlled by multisite phosphorylations; when intracellular calcium levels rise, calcium binds to calmodulin, which activates CAMK2 and leads to self-phosphorylation of CAMK2 at T286 47,48. Therefore, SYT12 may activate CAMK2 by decreased CAMK2N1 and increasing the intracellular calcium concentration in OSCCs, as a result, they promote the OSCC progression.

L-dopa is used widely to increase dopamine concentrations in the treatment of Parkinson's disease with a dopa decarboxylase inhibitor, such as carbidopa and benserazide, which inhibit aromatic amino acid decarboxylase, thus preventing the conversion of L-dopa into dopamine 49. L-dopa was recently reported to suppress cellular growth via down-regulation of prolactin-mediated JAK2/STAT5A in breast cancer cells 50, and carbidopa was reported to be an aryl hydrocarbon receptor agonist and to suppress the growth of pancreatic cancer 51. However, the role of L-dopa in oral cancer remains unknown. In the current study, we revealed for the first time that L-dopa led to down-regulation of SYT12 and decreased cellular proliferation by the cell cycle arrest at the G1 phase via p27^Kip1^.

In conclusion, we present here that SYT12 plays a significant role in OSCC progression via CAMK2N1 and CAMK2, and that L-dopa would be a new drug for OSCC treatment through the SYT12 expression.

## Supplementary Material

Supplementary figures.

## Figures and Tables

**Table 1:** Correlation between SYT12 expression and clinical classification in OSCCs.

Clinical classification		Results of immunostaining No. patients		
	Total	SYT12 negative	SYT12 positive	*P* value
Age at surgery (years)				
< 60	26	12	14	0.823
≧60	86	44	42	
				
				
Gender				
Male	66	32	34	0.706
Female	46	24	22	
				
T-primary tumor				
T1+T2	64	38	26	0.035^‡^
T3+T4	48	18	30	
			
N-regional lymph node				
Negative	74	43	31	0.028^‡^
Positive	38	13	25	
				
TNM Stage				
I+II	46	30	16	0.012^‡^
III+IV	66	26	40	
				
				
Vascular invasion				
Negative	88	48	40	0.106
Positive	24	8	16	
				
Histopathologic type				
Well	86	45	41	0.503
Moderately+Poorly	26	11	15	
				

Fisher's exact test, ^‡^*P* < 0.05.

## Abbreviations

SYT12: Synaptotagmin12
OSCC: oral squamous cell carcinoma
L-dopa: L-3, 4-dihydroxyphenylalanine
HNOKS: human normal oral keratinocytes
CAMK2N1: calcium/calmodulin-dependent protein kinase 2 inhibitor 1
CAMK2: calcium/calmodulin-dependent protein kinase 2
