# Supplementary material for: SYT12 plays a critical role in oral cancer and may be a novel therapeutic target
Source: J Cancer. 2019 Aug 27;10(20):4913–20. doi: 10.7150/jca.32582 (PMC6775516; doi:10.7150/jca.32582)

Supplementary Figure S1
(Quantification of the immunoblotting in Fig. 2E, 3C, 4C, and 4F.)

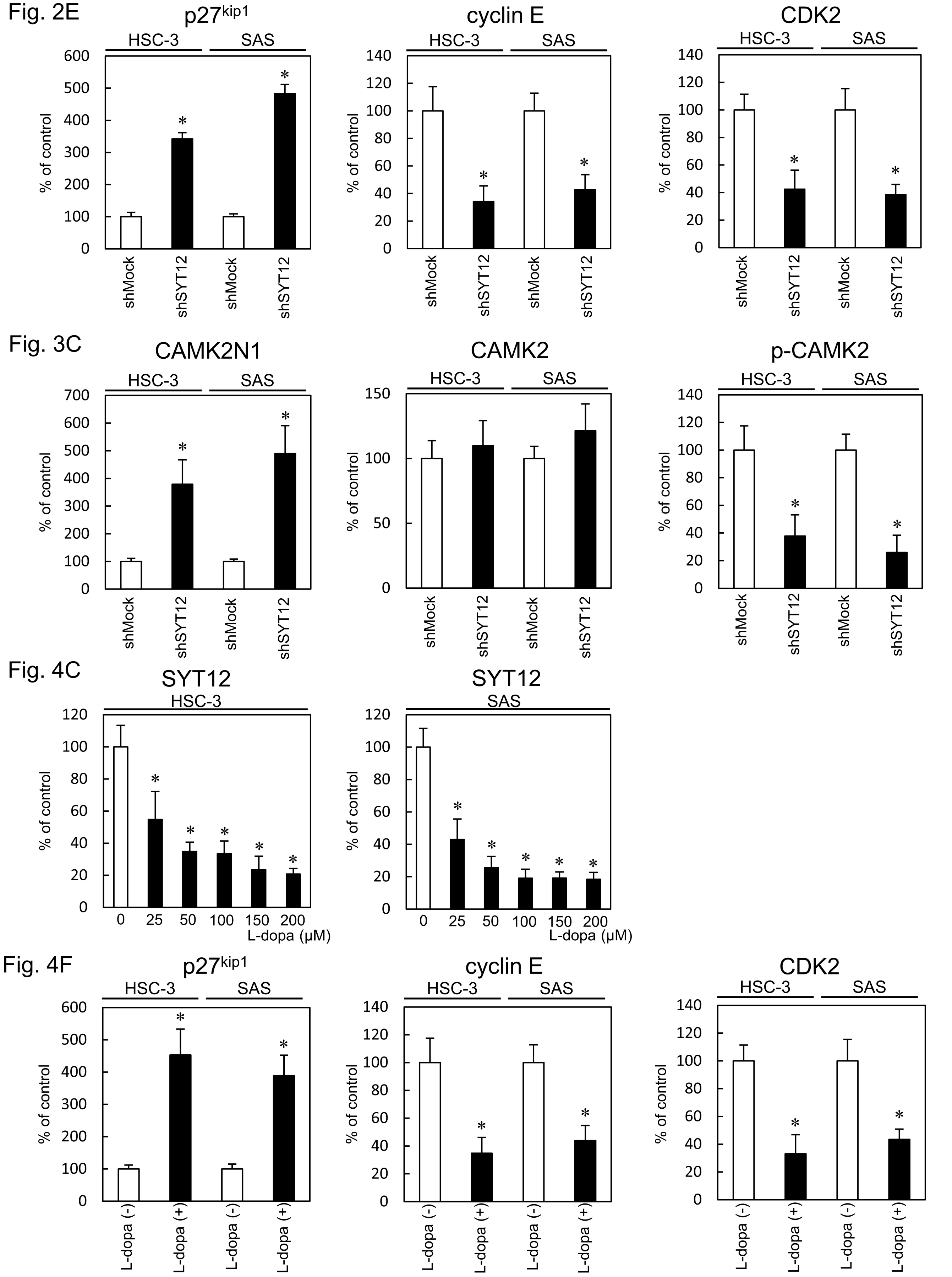

Supplementary Figure S2
(Full length blots of Fig.1B, 2B, 2E, 3C, 4C and 4F.)

Fig. 1B

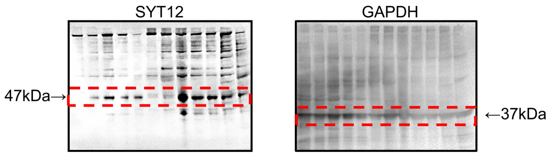

Fig. 2B

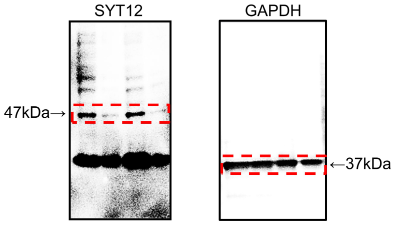

Fig. 2E

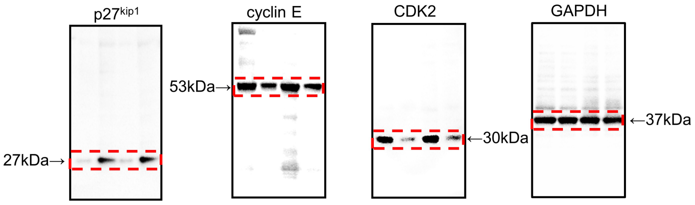

Fig. 3C

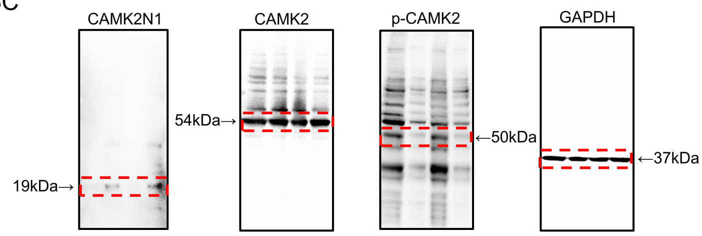

Fig. 4C

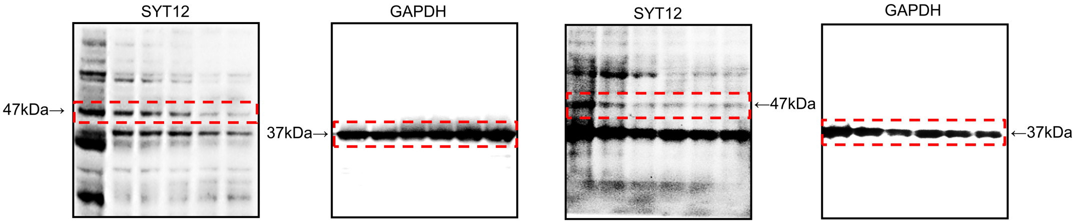

Fig. 4F

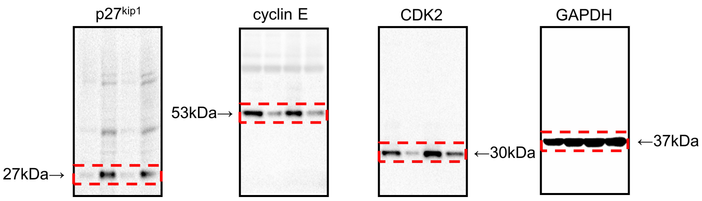

Supplement: Supplementary file 1 — Supplementary figures. [file jcav10p4913s1.pdf]
